# Excellent Islet Yields after 18-h Porcine Pancreas Preservation by Ductal Injection, Pancreas Preservation with MK Solution, Bottle Purification, and Islet Purification Using Iodixanol with UW Solution and Iodixanol with MK Solution

**DOI:** 10.3390/jcm8101561

**Published:** 2019-09-30

**Authors:** Kazuho Kuwae, Chika Miyagi-Shiohira, Eri Hamada, Yoshihito Tamaki, Kai Nishime, Mayuko Sakai, Tasuku Yonaha, Erika Makishi, Issei Saitoh, Masami Watanabe, Hirofumi Noguchi

**Affiliations:** 1 Department of Regenerative Medicine, Graduate School of Medicine, University of the Ryukyus, Okinawa 903-0215, Japan; e164157@eve.u-ryukyu.ac.jp (K.K.); chika@med.u-ryukyu.ac.jp (C.M.-S.); e144109@eve.u-ryukyu.ac.jp (E.H.); t97yo.shi328@gmail.com (Y.T.); e174183@eve.u-ryukyu.ac.jp (K.N.); e164202@eve.u-ryukyu.ac.jp (M.S.); e174153@eve.u-ryukyu.ac.jp (T.Y.); e154166@eve.u-ryukyu.ac.jp (E.M.); 2 Division of Pediatric Dentistry, Graduate School of Medical and Dental Science, Niigata University, Niigata 951-8514, Japan; isaito@dent.niigata-u.ac.jp; 3 Department of Urology, Okayama University Graduate School of Medicine, Dentistry and Pharmaceutical Sciences, Okayama 700-8558, Japan; masami5@md.okayama-u.ac.jp

**Keywords:** islet transplantation, islet isolation, islet purification, iodixanol, University of Wisconsin solution, extracellular-type trehalose-containing Kyoto (ETK) solution, modified ETK (MK) solution

## Abstract

Successful islet isolation is the key to successful islet transplantation. Our group recently modified the islet isolation protocol to include pancreatic ductal injection of the preservation solution, pancreas storage in modified extracellular-type trehalose-containing Kyoto (MK) solution, and use of an iodixanol-based purification solution and bottle purification. In this study, we applied these methods to porcine islet isolation after 18-h pancreas preservation and compared two solutions with different compositions in bottle purification. Islet yield before purification was 651,661 ± 157,719 islet equivalents (IE) and 5576 ± 1538 IE/g pancreas weight. An IU solution was made by adding iodixanol to University of Wisconsin solution and an IK solution was made by adding iodixanol to MK solution. The efficacy of the two solutions for islet isolation was compared. There were no significant differences between the two purification methods with regard to islet yield, survival rate, purity, score, or stimulation index. These results indicate that our isolation protocol produces efficient islet yields from prolonged cold-stored pancreas and that IU and IK solutions are equally useful for islet purification.

## 1. Introduction

Pancreatic islet transplantation is an excellent potential treatment for type 1 diabetic patients with difficulty controlling blood glucose levels or hypoglycemia unawareness despite maximal care [1,2,3,4,5]. While the transplantation procedure is simple and minimally invasive, the pancreatic islet isolation process requires considerable technical skill. The process is mainly composed of a collagenase injection, pancreatic digestion and islet purification. Islet purification is one of the most difficult and important processes for obtaining a high quantity of high-quality islets. The most common method for islet purification is density gradient centrifugation, which is based on the difference in density between pancreatic islets and acinar tissue [1,2,6,7]. Ficoll solution is the most commonly used solution for purification of pancreatic islets [1,2]. However, we previously reported that a controlled density gradient with iodixanol and organ preservation solution during islet purification leads to a better recovery rate than that with Ficoll solution in human islet isolation [6].

University of Wisconsin (UW) solution is commonly used for pancreas preservation. However, we previously showed that modified extracellular-type trehalose-containing Kyoto (ETK) solution significantly improved the islet yield compared to UW preservation for both 2- and 18-h preservation [8,9]. Modified ETK (MK) solution contains ulinastatin, a trypsin inhibitor, and has a high sodium/low potassium composition. Because the trypsin activity during pancreas preservation affects autolysis and the high potassium concentration induces insulin release from islets [10], MK solution is advantageous for the storage of pancreata for islet transplantation. Moreover, UW solution inhibits the activity of collagenases, an enzyme blend associated with pancreatic digestion [11,12], while MK solution inhibits collagenase activity to a lesser extent [9]. We have used MK solution in the preservation of pancreata for clinical islet transplantation by donation after brain death (DBD) and donation after cardiac death (DCD).

In the present study, we used purification solutions of iodixanol with UW solution (IU solution) and iodixanol with MK solution (IK solution) and compared their efficacy for islet purification. Given that the use of human pancreatic tissue from cadaveric donors for research is against the law in Japan, we used porcine pancreatic tissue in this study.

## 2. Experimental Section

### 2.1. Pancreatic Islet Isolation

Pancreata were removed from three-year-old porcine donors in a local slaughterhouse and a cannula was immediately inserted into the main pancreatic duct. The pancreas was weighed, and 1 mL/g pancreas weight of MK solution (ETK solution (Otsuka Pharmaceutical Factory, Naruto, Japan) with ulinastatin) was infused through the intraductal cannula [13]. Pancreata were then stored in chilled MK solution [9]. The “operation time” was defined as the time from the start of the operation until the removal of the pancreas. The warm ischemic time was defined as the time from the cessation of the animal’s heartbeat until placement of the pancreas into the preservation solution. The cold ischemic time, Phase I period, and Phase II period, were defined as described previously [4]

**Figure 1:**
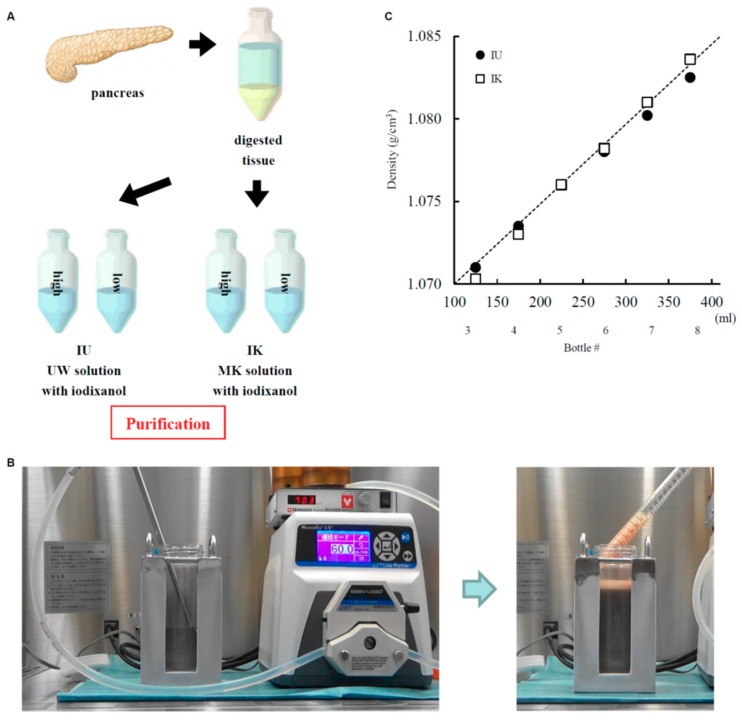
Density of the iodixanol to University of Wisconsin solution (IU) and iodixanol to modified extracellular-type trehalose-containing Kyoto solution (IK) gradients. (**A**) A schematic drawing of the islet purification method. (**B**) Pictures of bottle purification. (**C**) Theoretical density and calculated density of the IU and IK gradients. Dotted line: theoretical density; black circles: calculated density of gradient using IU solution; white squares: calculated density of gradient using IK solution.

To isolate the islets, the ducts were perfused in a controlled fashion with a cold enzyme blend of Liberase Mammalian Tissue Free (MTF) (1.0 mg/mL) with thermolysin (0.075 mg/mL) (Roche Diagnostics Corporation, Indianapolis, IN, USA). The islets were then separated by gentle mechanical dissociation [4,14] and purified using a continuous gradient of iodixanol-MK solution [6,15] or iodixanol-UW solution (Bridge to Life Ltd., Columbia, SC, USA) [16,17,18]. To generate new purification solutions, iodixanol was combined with UW solution (IU solution) or MK solution (IK solution) [6] (Figure 1A). We adopted bottle purification (size 500 mL; Nalgene, Rochester, NY, USA) in this step [18,19]. The digested tissue was divided in half so that equal amounts of tissue were used for each group. A gradient was generated using a gradient marker (Biorep Technologies, Miami Lakes, FL, USA) and candy cane-shaped stainless steel pipes (length 30 cm; UMIHIRA, Kyoto, Japan) to enable loading from the low-density solution to high-density solution, leaving the stainless steel pipe in place. After generating a continuous gradient, the digested tissue was loaded as the top layer [17] (Figure 1B). The bottles were centrifuged at 1000 rpm (235×g) for 5 min at 4 °C. After centrifugation, about 9 fractions (50 mL each) were collected and examined for purity.

### 2.2. Assessment of Islet Function

Dithizone (DTZ; Sigma-Aldrich, St. Louis, MO, USA) staining, scoring of gross morphology (score), and double fluorescein diacetate/propidium iodide (FDA/PI; Sigma-Aldrich) staining were performed as described previously [1,4,14,20]. The crude number of islets in each diameter class was determined by counting islets after DTZ staining using an optical graticule. The crude number of islets was then converted to the standard number of islet equivalents (IE; diameter standardized to 150 µm) [1,20]. Islet function was assessed by monitoring the insulin secretory response of the purified islets during glucose stimulation using the procedure described by Shapiro et al. [1,2]. The data were expressed as the mean ± standard error of the mean (SE).

### 2.3. Measurement of ATP Production

To measure the production of ATP, isolated islets in each group were cultured overnight with Connaught Medical Research Laboratories Medium (CMRL-1066; Sigma-Aldrich) supplemented with 0.5% human serum albumin (HSA; Sigma-Aldrich), washed twice with ice-cold phosphate buffered saline (PBS; Sigma-Aldrich), and solubilized. The amount of ATP was measured using an ATP assay system (Toyo Inki, Tokyo, Japan) according to the manufacturer’s instructions. Briefly, after allowing the reagents to equilibrate to room temperature, 10 μL of cell extracts were added to 100 μL of the reagents. The samples were measured using a luminometer.

### 2.4. In Vivo Assessment

Isolated islets were incubated for 6 h. A total of 2000 IE of the porcine islets obtained from each group were processed for transplantation. Diabetes induction, transplantation into nude mice (six-week-old, male; Charles River Laboratories Japan, Inc., Kanagawa, Japan) (*n* = 20), and intraperitoneal glucose tolerance testing (IPGTT) were performed as described previously [21,22,23]. All animal studies were approved by the Institutional Animal Care and Use Committee of the University of the Ryukyus.

### 2.5. Statistical Analyses

The data are expressed as the mean ± SE. Differences between the two groups were analyzed using Student’s t-test or the Kaplan-Meier log-rank test. *P* values of <0.05 were considered statistically significant.

## 3. Results

### 3.1. Characteristics of the Isolated Porcine Islets

**Figure 2:**
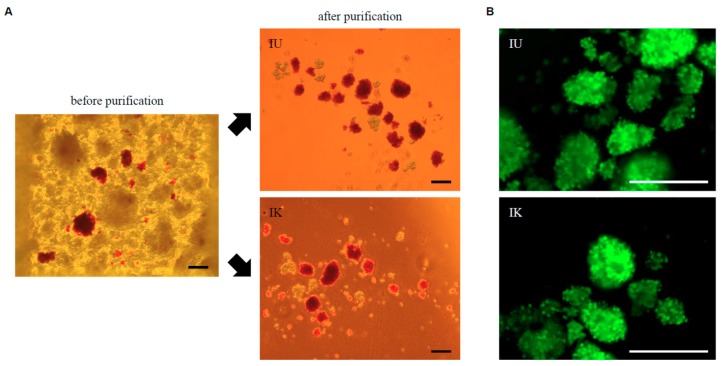
Dithizone (DTZ) and fluorescein diacetate/propidium iodide (FDA/PI) staining of islets. (**A**) DTZ staining before and after islet purification. (**B**) FDA/PI staining of isolated islets. Scale bars = 200 μm.

Islet isolation was conducted as described previously [4] according to the standard Ricordi technique [3] with modifications later introduced in the Edmonton protocol [1,2]. The characteristics of the porcine pancreata and islets before purification are summarized in Table 1. Islet yield before purification was 651,661 ± 157,719 IE and 5576 ± 1538 IE/g pancreas weight. The digested tissue was incubated in UW solution before purification. Islets were purified using a continuous density gradient of IU solution or IK solution (Figure 1A). We combined iodixanol with UW solution or MK solution to produce the IU and IK purification solutions, respectively. Low-density (1.075 g/cm^3^) and high-density (1.085 g/cm^3^) solutions were produced by changing the volumetric ratio of iodixanol and each preservation solution, as shown in Table 2. The theoretical density and calculated density of each continuous gradient are shown in Figure 1C. The digested tissue was divided in half so that equal amounts of tissue were used for each group. There were no significant differences in islet yield after purification (IU group: 276,719 ± 64,342 IE, 2375 ± 631 IE/g; IK group: 271,875 ± 47,910 IE, 2294 ± 487 IE/g; Table 3), or in the post-purification recovery rate (Table 3), purity (Figure 2A, Table 4), viability (Figure 2B, Table 4), or score (Table 4). These data suggest that the two solutions had a similar level of efficacy for islet purification.

### 3.2. In Vitro Assessment

To assess the quality of the islets in each group in vitro, we measured the stimulation index of the isolated islets. There were no significant differences in the stimulation index between islets isolated using the two solutions (IU group: 1.34 ± 0.22, *n* = 5; IK group: 1.32 ± 0.38, *n* = 5; Table 3). The adenosine triphosphate (ATP) concentration of the cell lysate after islet isolation was measured in each group using an ATP assay system. The ATP content was similar between the two groups (*n* = 10 each) (IU group: 0.85 ± 0.04 pmol/IE; IK group: 0.85 ± 0.05 pmol/IE; Table 3). These data suggest that the islets in the two groups were of similar quality in vitro.

### 3.3. In Vivo Assessment

**Figure 3:**
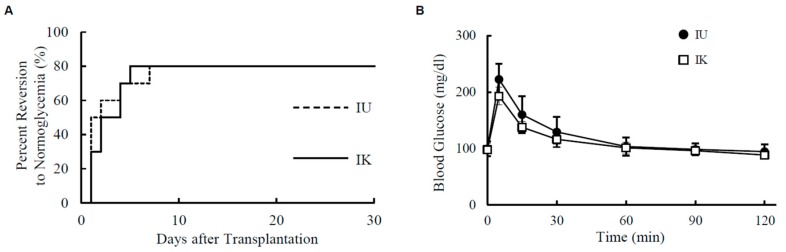
Islet transplantation into diabetic nude mice. (**A**) The percentage of streptozotocin (STZ)-induced diabetic nude mice in which normoglycemia was achieved after islet transplantation is shown. A total of 2000 IEs were transplanted below the kidney capsule of the diabetic nude mice. Normoglycemia was defined as two consecutive post-transplant blood glucose level measurements of <200 mg/dL (IU group, *n* = 10; IK group, *n* = 10). (**B**) The results of the intraperitoneal glucose tolerance testing (IPGTT). Normoglycemic mice at 30 days after islet transplantation were fasted overnight and then intraperitoneally injected with glucose (2.0 g/kg body weight). Blood glucose levels were measured before and at 5, 15, 30, 60, and 120 min after glucose injection (IU group, *n* = 5; IK group, *n* = 5).

To evaluate the quality of islets in the IU group and IK group in vivo, 2000 IEs from each group were transplanted into diabetic nude mice induced by streptozotocin (STZ). In the in vivo transplantation model, the curative rate was the same between the IU group (8 of 10 mice (80.0%)) and IK group (8 of 10 mice (80.0%)) (Figure 3A). IPGTT was also similar between the IU group and IK group (Figure 3B). These data suggest that the quality of isolated islets in the two groups was comparable in vivo.

## 4. Discussion

Restoration of pancreas endocrine function is potentially and completely achieved using pancreatic islet transplantation in patients with type 1 diabetes with extreme glycemic variability [1,2,3,4,5]. Although numerous improvements in islet isolation have led to much higher islet yields, islets are still lost during purification. Several purification techniques have been reported, including magnetic retraction [24], filtration [25], antiacinar cytotoxic antibodies [26], isopycnic density gradient centrifugation using the COBE 2991 cell processor (COBE) [27], and bottle purification [18,19]. The development of gradient media has improved islet purification recovery by density gradient centrifugation [28,29,30].

Previous studies have reported that MK solution is superior to UW solution for both 2- and 18-h preservation of pancreatic islets [8,9]. However, we found that IK solution was similarly effective to IU solution for islet purification in this study. There are two possible reasons for the discrepancy in results. UW solution has several disadvantages, including inhibition of the activity of collagenases, enzymes used for pancreatic digestion [11,12]. In contrast, MK solution has several advantages, including trypsin inhibition by ulinastatin and lower collagenase inhibition, which does not decrease the energy levels retained in the preserved pancreas [8,9]. However, pancreatic islets are only in contact with each purification solution for 10 to 20 min, which is too short a time for the preservation solution to have any marked effects on the islet cells. Moreover, collagenase inhibition is not advantageous for purification because collagenases are not used in this step. Therefore, the features of MK solution that make it advantageous for the preservation step are worthless for the purification step.

In this study, the cold ischemic time was approximately 1100 min, which is long for islet isolation. Our data represent an excellent outcome despite this prolonged cold ischemic time, and may be due to our modifications of the Ricordi/Edmonton islet isolation methods. These modifications included pancreatic ductal injection of the preservation solution [13], pancreas preservation with MK solution [9], and use of an iodixanol-based purification solution [6,15,16,17,18] and bottle purification [16,17,18]. We have successfully performed clinical islet transplantation using pancreata from not only DBD [31] but also DCD [4] and living donor [32] using our islet isolation method.

## 5. Conclusions

Our data show that this method offers excellent porcine islet isolation using prolonged cold-stored pancreas. IU and IK solutions had similar efficacy for islet purification. These solutions are equally useful as islet purification solutions for pancreatic islet purification.

## Figures and Tables

**Table 1:** Characteristics of the tissue and procedures before purification.

Characteristics of the Tissue and Procedures	*n* = 5
Pancreas weight (g)	124.3 ± 8.9
Operation time (min)	4.2 ± 0.6
Warm ischemic time (min)	26.4 ± 0.8
Cold ischemic time (min)	1100.8 ± 16.5
Phase I period (min)	11.2 ± 0.8
Phase II period (min)	39.4 ± 0.5
Undigested tissue (g)	11.4 ± 1.3
Islet yield before purification (IE)	651,661 ± 157,719
Islet yield before purification (IE/g)	5576 ± 1538

The data are expressed as the mean ± SE.

**Table 2:** Characteristics of each purification solution.

**IU Solution**	**UW (mL) ^a^**	**Iodixanol (mL) ^b^**	**Final Density (g/cm^3^)**
High density	500	78.7	1.0850
Low density	500	55.1	1.0750
**IK Solution**	**MK (mL) ^c^**	**Iodixanol (mL) ^b^**	**Final Density (g/cm^3^)**
High density	500	100	1.0850
Low density	500	75.5	1.0750

^a^ Density of UW solution is 1.048 g/cm^3^. ^b^ Density of iodixanol solution is 1.320 g/cm^3^. ^c^ Density of MK solution is 1.038 g/cm^3^.

**Table 3:** Characteristics of islets after purification.

**IU**	**Solution**							
#	IE	IE/g	% Recovery ^a^	Insulin content ^b^	Low glucose ^b^	High glucose ^b^	SI ^c^	ATP ^d^
1	98,620	721	77.1	382	10.7	12.6	1.18	0.71
2	407,996	3699	77.0	483	15.8	30.8	1.95	0.93
3	427,452	3730	87.4	458	16.1	27.3	1.69	0.91
4	171,284	1117	83.4	383	14.2	9.9	0.70	0.83
5	278,242	2605	100.5	480	13.8	16.3	1.18	0.88
Ave	276,719	2375	85.1	437	14.1	19.4	1.34	0.85
SE	64,342	631	4.3	23	1.0	4.1	0.22	0.04
**IK**	**Solution**							
#	IE	IE/g	% Recovery ^a^	Insulin content ^b^	Low glucose ^b^	High glucose ^b^	SI ^c^	ATP ^d^
1	117,754	861	92.0	372	11.8	11.9	1.01	0.66
2	355,868	3226	67.1	453	15.2	42.2	2.77	0.97
3	388,156	3387	79.4	468	15.9	18.8	1.18	0.93
4	232,816	1519	113.4	392	14.8	8.9	0.60	0.85
5	264,780	2479	95.6	489	14.2	14.6	1.03	0.85
Ave	271,875	2294	89.5	435	14.4	19.3	1.32	0.85
SE	47,910	487	7.8	23	0.7	6.0	0.38	0.05

**^a^** Post-purification recovery (%) = islet equivalents (IE) after purification/(IE before purification/2) × 100. **^b^** pg/ng Protein, **^c^** Stimulation index, **^d^** pmol/IE. #: number.

**Table 4:** Viability, purity, and score of islets after purification.

Characteristics of Islets	IU (*n* = 5)	IK (*n* = 5)
Viability (%)	96.0 ± 0.5	95.5 ± 0.4
Purity (%)	70.0± 5.1	72.0 ± 4.6
Score	9.6 ± 0.4	9.6 ± 0.1

The data are expressed as the mean ± SE.

## Abbreviations

ETK	extracellular-type trehalose-containing Kyoto
MK	modified ETK
UW	University of Wisconsin
IU	iodixanol with UW
IK	iodixanol with MK
DBD	donation after brain death
DCD	donation after cardiac death
ATP	adenosine triphosphate
STZ	streptozotocin
RPMI	Roswell Park Memorial Institute
IEs	islet equivalents
FDA/PI	fluorescein diacetate/propidium iodide
SE	standard error
CMRL	Connaught Medical Research Laboratories Medium
HSA	human serum albumin
PBS	phosphate buffered saline
IPGTT	intraperitoneal glucose tolerance testing
